# Comparison of steam technology and a two-step cleaning (water/detergent) and disinfecting (1,000 resp. 5,000 ppm hypochlorite) method using microfiber cloth for environmental control of multidrug-resistant organisms in an intensive care unit

**DOI:** 10.3205/dgkh000330

**Published:** 2019-10-24

**Authors:** Nefise Oztoprak, Filiz Kizilates, Duygu Percin

**Affiliations:** 1 Antalya Education and Research Hospital, Department of Infectious Diseases and Clinical Microbiology, Antalya, Turkey; 2 Kutahya Health Sciences University Medical Faculty, Department of Medical Microbiology, Kutahya, Turkey

**Keywords:** multidrug-resistant bacteria, hospital cleaning, steam technology, disinfectants, hypochlorite solutions, cost analysis, environmental cleaning

## Abstract

**Aim:** The aim of this prospective observational study was to evaluate the impact of two cleaning and disinfecting methods and the use of steam against methicillin-resistant *Staphylococcus aureus*, vancomycin-resistant *Enterococcus faecalis*, carbapenem-resistant *Pseudomonas aeruginosa* and multidrug-resistant (MDR) *Acinetobacter baumannii* in a tertiary referral hospital.

**Methods:** McFarland 0.5 suspensions (content 1.5 x 10^8^ cfu/ml) of four challenge bacterial species were prepared and used to inoculate different sites in three ICU rooms. One of the following methods was used in each room: steam technology (Tecnovap Evo 304) resp. cleaning with microfiber cloths, soaked with detergent and water, thereafter disinfection with 1,000 ppm hypochlorite or the same procedure with 5,000 ppm hypochlorite. Qualitative microbiology and ATP bioluminescence were performed before and after cleaning with each method. The Wilcoxon test was used for paired samples to check for ordinal variables. The cost of each cleaning method was analyzed.

**Results:** Environmental cleaning with steam technology was found to be as effective against MDR microorganisms as a two-step cleaning process (water/detergent and disinfecting with 1,000 resp. 5,000 ppm hypochlorite) in ICUs. No bacterial growth was detected after any of the three cleaning methods. Steam technology was 76% and 91% cheaper than using 5,000 ppm and 1,000 ppm hypochlorite, respectively.

**Conclusions:** When compared to, steam technology was found to have an advantage over the 2-step procedure with cleaning and disinfection, because it avoids the use of chemicals, reduces water consumption, labor time and costs for cleaning.

## Zusammenfassung

**Zielsetzung:** In einer prospektiven Beobachtungsstudie sollte die Wirksamkeit von zwei Verfahren der desinfizierenden Reinigung bzw. der Anwendung von Dampf gegen Methicillin-resistente *Staphylococcus aureus* (MRSA), Vancomycin-resistente *Enterococcus faecalis* (VRE), Carbapenem-resistente *Pseudomonas aeruginosa* und multiresistente (MDR) *Acinetobacter baumannii* in einem Krankenhaus der Tertiärversorgung verglichen werden.

**Methode:** Von den vier Bakterienspecies wurden Suspensionen mit einem Gehalt von 1,5 x 10^8^ KbE/ml hergestellt und zur Kontamination von drei Lokalisationen in drei Intensivstationen eingesetzt. In jedem Raum wurde eine der folgenden Reinigungsmethoden angewendet: Dampftechnologie (Tecnovap Evo 304) oder 2 Formen der desinfizierenden Reinigung in zwei Schritten: zuerst Reinigung mit Tensid getränktem Mikrofasertuch, anschließend Wischdesinfektion mit 1.000 ppm oder analoges Vorgehen nur mit 5.000 ppm Hypochlorit. Vor und nach der desinfizierenden Reinigung wurde ein qualitativer Erregernachweis geführt und die Reinigungswirkung mittels ATP-Biolumineszenz gemessen. Der Wilcoxon-Test wurde für gepaarte Stichproben zur Überprüfung ordinaler Variablen verwendet. Die Kosten für jede Reinigungsmethode wurden analysiert.

**Ergebnisse:** Die drei Verfahren erwiesen sich als gleich wirksam. Nach keiner der drei Reinigungsmethoden war ein Bakterienwachstum nachweisbar. Die Dampftechnologie war 76% bzw. 91% preiswerter als die Verwendung von 5.000 ppm bzw. 1.000 ppm Hypochlorit.

**Schlussfolgerung:** Im Vergleich zur zweistufigen desinfizierenden Reinigung mit Chlorlösungen erwies sich die Dampftechnologie als vorteilhafter, da auf den Einsatz von Chemikalien verzichtet und der Verbrauch von Wasser, benötigter Arbeitszeit und Reinigungskosten reduziert wird.

## Introduction

Environmental decontamination is critical, particularly in ICUs, for the prevention and control of healthcare-associated infections (HAIs). There is strong evidence that contaminated environmental surfaces transmit pathogens in healthcare settings [1], [2]. However, traditional cleaning methods include a range of detergents and disinfectants. The use of surface disinfectants is controversial for safety reasons, as most disinfectants are toxic to humans or cause allergies [3], [4]. 

Liquid chemical disinfectants such as sodium hypochlorite, quaternary ammoniums and hydrogen peroxide are those usually used for environmental decontamination [5]. Chlorine solutions, such as sodium hypochlorite solutions or solutions prepared with sodium dichloroisocyanurate tablets – which release active chlorine with potent germicidal action – are recommended for surface disinfection in hospitals [4]. These solutions are cheap and fast acting, and remove dried or fixed organisms and biofilms from surfaces [6], [7]. They also have a broad spectrum of antimicrobial activity, which is unaffected by water hardness. However, the pH of the water used for diluation greatly affects efficacy [6], [8]. Nevertheless, sodium hypochlorite has a low incidence of serious toxicity, although it can produce ocular irritation, oropharyngeal, esophageal and gastric burns [9], [10]. 

Newer technologies for environmental cleaning are now becoming available. Furthermore, these technologies can only be used for terminal or discharge cleaning because the products are either toxic for patients (e.g., hydrogen peroxide) or are better suited to work within unoccupied rooms (e.g., UV light). Steam technology and microfiber cloths/mops has proven to be a good choice for environmental cleaning in healthcare settings in recent years [5], [11], [12], [13]. It is not toxic for humans so it can be used even in occupied rooms. It is used for cleaning both hard surfaces and textiles. This technology poses no risk on the environment, as no chemicals are used. It not only saves time and costs, it also reduces water use by about 90%. Steam technology uses superheated dry steam (<140°C) delivered under pressure to loosen dirt and sticky oils from surfaces. The high temperature of the steam kills microorganisms. Steam cleaning machines first apply steam to the surface and then use vacuum suction, removing dirt, water and contaminants from the area being cleaned [14]. There are many benefits to using steam in both routine and outbreak situations, even for biofilms on surfaces [5], [8], [11], [12], [15], [16]. 

Evaluating the quality of different cleaning regimens still has been limited. There are only visual methods, microbiological testing, adenosine triphosphate (ATP) monitoring and ultraviolet visible markers (UVM) that report the presence of contamination [17], [18]. Visual assessment of cleaning can be misleading, because it can be subjective. For evaluation of cleanliness in contrast to conventional microbiological testing, which requires almost two days, some rapid methods exist, such as MALDI-TOF, as well as ATP and UVM, which provide results within minutes after sampling. Rapid ATP is a commonly used method to measure cleanliness on reusable medical devices and healthcare environmental surfaces [19]. It has been suggested as a quantitative method of assessing surface hygiene that is superior to simple visual inspection [20]. Rapid ATP testing devices are simple to use, lightweight and portable, provide an almost immediate reading, and the consumables required are relatively inexpensive. ATP testing devices measure all cellular ATP and not just microbial ATP; thus, it is a broad indicator of cleanliness from all biological concomitants and useful for cleanliness monitoring [17], [21]. A new ATP sampling algorithm was designed to reduce the impact of inherentt variability and imprecision on any individual sampling surface or medical device. It provides a superior level of certainty with field-based ATP data through the mitigation of inherent device imprecision and variability [19]. ATP testing is not a substitute for microbiological testing of surfaces, although a number of studies have indicated the value of rapid ATP testing for cleanliness monitoring and training within healthcare settings [22], [23]. 

The aims of our study were to evaluate the cleaning efficacy of the tested methods against methicillin-resistant *Staphylococcus aureus* (MRSA), vancomycin-resistant *Enterococcus faecalis* (VRE), carbapenem-resistant *Pseudomonas aeruginosa* and MDR *Acinetobacter baumannii* by microbiological testing and ATP monitoring, and to assess the cost effectiveness of the methods in an ICU. 

## Methods

This study was done in a tertiary referral health service offering hospitalization to about 71,371 patients every year, where an average of 2,790 ICU patients are treated per year, with a total of 43 ICU beds. It was conducted in September 2015 in three separate patient rooms (one bed per room) in a 12-bed resuscitation ICU. Two-step cleaning is done at our ICUs, which consists of first cleaning with detergent and water with microfiber cloths, followed by a second step of disinfection with a sodium hypochlorite solution. Hypochlorite solutions were prepared with sodium dichloroisocyanurate tablets. Cleaning was performed three times per day using 5,000 ppm hypochlorite solution in rooms where patients with infection and/or colonization with MDR microorganisms stayed; however it was performed twice per day with 1,000 ppm hypochlorite solutions in all other patient rooms at our hospital. 

The challenge (test) bacterial suspensions of *Staphylococcus aureus* ATCC 43300 (methicillin-resistant), *Enterococcus faecalis* ATCC 51299 (vancomycin-resistant), *Pseudomonas aeruginosa* ATCC BAA-2108 (carbapenem-resistant) and *Acinetobacter baumannii* ATCC 19606 (MDR) were prepared at 0.5 McFarland concentrations by subculturing twice. 

In rooms where the studies were conducted, two-step cleaning was performed first. After this standard cleaning procedure the pre-prepared bacterial suspensions (0.1 mL) were then applied to surface areas of 0.5x0.5 cm at five test sites for each bacterial species and left to dry for 10 minutes. The test sites included monitor buttons (plastic), bedside tables (medium density fiberboard), bed remote controls (plastic), bed rails (plastic) and floor (polyvinyl chloride). After this standardized bacterial contamination swab samples were taken both for microbiological culture and ATP determination before the study cleaning processes. The first room was disinfected with 1,000 ppm hypochlorite solution, the second room was disinfected with 5,000 ppm hypochlorite solution, and the third room was disinfected with steam technology. However in the first and second room two-step cleaning with microfiber cloths/mops was performed; in the third room only steam disinfection was performed. The steam disinfection device (Tecnovap Evo 304, Tecnovap, Italy) was used for 10 seconds contact time using 8-bar steam at a temperature of 174°C. Tap water was used for this and no disinfectant was added. 

Samples were taken with sterile swabs from the same contaminated sites for the second time both for microbiological culture and ATP from the three rooms after the process. The contamination was measured in Relative Light Units (RLU) using a bioluminescence monitor (Clean-Trace ATP System, NG Luminometer; 3M™ Microbiology), in accordance with the manufacturer’s instructions and literature [17], [18], [19]. The recommended level of cleanliness of <100 RLU is accepted as threshold for cleaning. ATP results were available within 10 seconds after sampling. The biological load of these sites after the respective procedures was also measured using microbiological sampling and culture. The samples taken for microbiological culture were inoculated onto agar plates containing 5% sheep blood and incubated aerobically for 48 hours at 37°C; CFUs were counted at the end of the incubation period. 

The cost analysis of cleaning methods included detergent, chlorine tablets, microfiber cloth and mop, water, electricity and the cost of the steam disinfection device. The steam device has a warranty of two years (730 days), and the cleaning time is 10 seconds for a bed. One device would be enough for 43 ICU beds. Therefore, the daily cost of a device was found by dividing 730 days by 43 beds. The cost of a microfiber cloth and microfiber mop was by the number used. Fifteen microfiber cloths and one microfiber mop were used per bed during one cleaning shift, and one microfiber cloth and mop can be reused nearly 30 times. The cost of hypochlorite solutions were assessed based on the number of chlorine tablets used. Cleaning staff costs were not included in the cost analysis. 

Data were analyzed using the Statistical Package for Social Science (SPSS) version 22 (SPSS Inc, Chicago, USA). The Wilcoxon test was used for paired samples to check for ordinal variables and p≤0.05 was considered statistically significant.

## Results

Both steam technology and hypochlorite solutions (1,000 ppm and 5,000 ppm) showed similar efficacy against MRSA, VRE, carbapenem resistant *Pseudomonas aeruginosa* and MDR *Acinetobacter baumannii*. No bacterial growth was detected after any of the tested cleaning methods. ATP results were consistent with microbiological culture results before and after the cleaning procedures. The mean ATP results of the contaminated surfaces before and after cleaning with all three cleaning methods are summarized in Table 1 (Tab. 1). Although all three methods were found to be effective for cleaning (all ATP readings were <100 RLU after cleaning), the ATP readings after cleaning with steam technology were significantly lower than with 1,000 ppm and 5,000 ppm hypochlorite solutions (p<0.05).

Cleaning with steam technology was able to penetrate surfaces without causing any damage and could reach relatively inaccessible areas like crevices by using the vacuum system to remove dirt and grime. But this method left some residual moisture, so the use of a microfiber cloth and mop was essential to dry the surface. While the steam method took 10 minutes, the chlorine cleaning methods took nearly 20 minutes for each ICU bed. Therefore, steam technology was more time-saving than cleaning with chlorine solutions. 

The cleaning cost per ICU bed was assessed at $0.91 using 5,000 ppm hypochlorite; $2.44 using 1,000 ppm hypochlorite and $0.22 using steam technology. Steam technology is 76% and 91% cheaper than using 5,000 ppm and 1,000 ppm hypochlorite, respectively. Steam technology was time-saving and also incurred nearly 50% less cleaning staff cost, although cleaning staff cost was not considered in this analysis. 

Water consumption with two-step cleaning using hypochlorite solutions was nearly 33 liters, whereas it was three liters using steam technology. Steam technology cut water consumption by nearly 91%. Cleaning staff delivered positive feedback, since there was no contact with chemicals and no chlorine odor. We did not detect any breathing problems from vapor inhalation or burns among the cleaning staff. 

## Discussion

HAIs are observed very frequently especially in ICUs [24]. Some important pathogens such as MRSA, VRE and MDR Gram-negative bacilli persist for days in the healthcare environment [25], [26]. These pathogens are frequently shed by patients and healthcare workers (HCWs), so they contaminate surfaces and increase the risk of transmission to other patients [27], [28]. About 20–40% of HAIs are transmitted by the hands of HCWs, either by direct contact with the patient or by touching contaminated surfaces [15]. It is known that environmental contamination is associated with the colonization and infection of ICU patients [29], [30]. When surfaces such as bed rails are contaminated, transmission starts with the hands of the HCWs and then spreads from patient to patient. Therefore, surface disinfection is essential at hospitals [31]. In a study of an *Acinetobacter baumannii* outbreak, it was shown that the infection would have been preventable with environmental cleaning [16].

There is increasing awareness about the role of cleaning for managing HAIs [32]. Hospital cleaning becomes a focus for patients, hospital managers and health authorities. It is supported by burgeoning research which has found that enhanced cleaning and decontamination during routine and costly outbreak situations is beneficial [33], [34]. Conventional cleaning based on detergent and disinfectant can help to control HAIs, although the cleaning procedure itself is subject to discussion about frequencies, methods, equipment, monitoring, and standards for surface hygiene around the world [35], [36]. 

As is well known, most chemical disinfectants are toxic to humans and long-term contact with these chemicals affects the health of HCWs. Hypochlorites are the most widely used chlorine disinfectant in environmental cleaning in ICUs [4]. They are available as liquid (e.g., sodium hypochlorite) or solid forms (e.g., sodium dichloroisocyanurate tablets). It is shown that they are effective even against *C. difficile* and *Norovirus*, which cause environmental contamination in ICUs [8]. Although chlorine solutions are inexpensive, they may cause ocular, oropharyngeal or gastric irritation. Other disadvantages of hypochlorites include corrosiveness to metals in high concentrations (>500 ppm), inactivation by organic matter, discoloring or “bleaching” of fabrics, release of toxic chlorine gas when mixed with ammonia or acid, and relative instability [37], [38], [39]. 

Beside conventional methods for environmental cleaning at hospitals, the efficacy of newer methods such as hydrogen peroxide, steam technology and use of ultra-microfiber polyester and polyamide cloths, which clean the particles with absorption and electrostatic action, is currently being studied [5], [11], [12], [15], [40], [41]. Hydrogen peroxide can be used in critical areas such as ICUs and operating rooms, but it is somewhat limited when used in the form of a toxic fog. Before people can re-enter the area, the space must be ventilated for a while after disinfection [42]. Therefore, it is not a convenient method for ICUs. The new steam technology is an effective means of environmental decontamination [5], [11], [12], [15]. Because only tap water is used in the steam disinfection method, it does not contain any corrosive or toxic materials, and it is applicable even in areas where patients are present, this method is more advantageous compared to hydrogen peroxide. As a rule, routine two-step environmental cleaning is performed at least twice a day at hospitals; however, in units such as ICUs where the infections with MDR microorganisms are frequent, it may be performed up to three times daily. Also, the hypochlorite concentrations used may be higher than normal. Steam disinfection eliminates the use of chemicals, is odourless, does not need extra ventilation and therefore is more advantageous compared to chlorine disinfectants. While heavy soiling must always be removed before chlorine disinfection, steam can be directly applied onto a wide variety of soft and hard surfaces without prior cleaning. Two-step cleaning is time consuming, and many surfaces do not tolerate chlorine solutions, including cloth drapes or chairs, carpets, or stethoscopes. Less cleaning time is another advantage of steam technology. Saving time also means a reduction in the number of cleaning staff. The other advantage of steam technology is that it consumes 90% less water. 

The present study has demonstrated that steam technology is as effective against MDR microorganisms as hypochlorite disinfectants in ICU environmental cleaning. We observed that cleaning with steam technology penetrated surfaces without causing any damage. Steam cleaning was able to reach inaccessible areas that conventional cleaning methods were not. It is well suited to cleaning heavily contaminated surfaces and equipment components. This can prevent microorganisms becoming fixed to environmental surfaces in ICUs. 

However, surfaces were damp after using steam technology and drying of the cleaned surfaces was necessary. Although the literature mentions that inhalation of the vapor could potentially aggravate breathing problems in staff or patients with respiratory conditions [42], we did not detect any breathing problems in patients or staff. Furthermore, it should be carefully used for electrical devices, for example ventilator push buttons, and keyboards.

In the present study, we used ATP monitoring along with conventional microbiological culturing methods, i.e., bacterial colony counting to evaluate the efficacy of environmental disinfection. ATP monitoring, which assesses the biological load in the environment in RLU, has been previously used by Gillespie et al. and proved to be effective [43]. ATP monitoring results were consistent with conventional microbiological results in the present study as well. ATP monitoring is a practical method for evaluating environmental cleaning in units such as ICUs, as it provides fast results and is easy to use, although it is relatively expensive.

Evaluating the cost effectiveness of cleaning methods is very difficult because environmental data are not usually modeled against patient outcome. We found that steam technology is 91% and 76% cheaper than 1,000 ppm or 5,000 ppm hypochlorite, respectively, for the control of MDR microorganisms. 

In conclusion, the present study confirmed that environmental cleaning with steam technology and microfiber cloths/mops was found to be as effective against methicillin-resistant *Stapylococcus aureus* (MRSA), vancomycin-resistant *Enterococcus faecalis* (VRE), carbapenem-resistant *Pseudomonas aeruginosa* and multidrug-resistant (MDR) *Acinetobacter baumannii* as cleaning with hypochlorite solutions in ICUs. Our data suggest that the steam technology is more cost effective, time saving and environmentally friendly than conventional cleaning methods. Quality improvement efforts should be made to improve environmental cleaning at hospitals, especially for MDR microorganism infections.

## Notes

### Competing interests

The authors declare that they have no competing interests.

## Figures and Tables

**Table 1:**
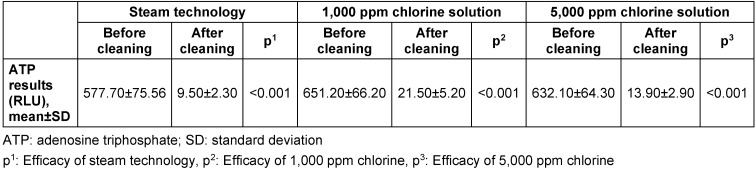
Adenosine triphospate results before and after cleaning procedures
